# Data-driven models for the prediction of coronary atherosclerotic plaque progression/regression

**DOI:** 10.1038/s41598-024-51508-7

**Published:** 2024-01-17

**Authors:** Carlos A. Bulant, Gustavo A. Boroni, Ronald Bass, Lorenz Räber, Pedro A. Lemos, Héctor M. García-García, Pablo J. Blanco

**Affiliations:** 1 Instituto PLADEMA, Universidad Nacional del Centro de la Provincia de Buenos Aires (UNICEN), Tandil, Buenos Aires, Argentina; 2 Consejo Nacional de Investigaciones Científicas y Técnicas (CONICET), Tandil, Buenos Aires, Argentina; 3 Georgetown University School of Medicine, Washington, D.C., USA; 4 Department of Cardiology, Inselspital, Bern University Hospital, University of Bern, Bern, Switzerland; 5 Heart Institute, University of São Paulo Medical School, São Paulo, SP Brazil; 6 Hospital Israelita Albert Einstein, São Paulo, SP Brazil; 7 Division of Interventional Cardiology of MedStar Cardiovascular Research Network, MedStar Washington Hospital Center, 110 Irving Street, Suite 4B-1, Washington, D.C., 20010 USA; 8 National Laboratory for Scientific Computing (LNCC-MCTI), Petrópolis, RJ Brazil; 9 National Institute of Science and Technology in Medicine Assisted by Scientific Computing (INCT-MACC), Petrópolis, RJ Brazil

**Keywords:** Atherosclerosis, Biomedical engineering, Applied mathematics, Information technology

## Abstract

Coronary artery disease is defined by the existence of atherosclerotic plaque on the arterial wall, which can cause blood flow impairment, or plaque rupture, and ultimately lead to myocardial ischemia. Intravascular ultrasound (IVUS) imaging can provide a detailed characterization of lumen and vessel features, and so plaque burden, in coronary vessels. Prediction of the regions in a vascular segment where plaque burden can either increase (progression) or decrease (regression) following a certain therapy, has remained an elusive major milestone in cardiology. Studies like IBIS-4 showed an association between plaque burden regression and high-intensity rosuvastatin therapy over 13 months. Nevertheless, it has not been possible to predict if a patient would respond in a favorable/adverse fashion to such a treatment. This work aims to (i) Develop a framework that processes lumen and vessel cross-sectional contours and extracts geometric descriptors from baseline and follow-up IVUS pullbacks; and to (ii) Develop, train, and validate a machine learning model based on baseline/follow-up IVUS datasets that predicts future percent of atheroma volume changes in coronary vascular segments using only baseline information, i.e. geometric features and clinical data. This is a post hoc analysis, revisiting the IBIS-4 study. We employed 140 arteries, from 81 patients, for which expert delineation of lumen and vessel contours were available at baseline and 13-month follow-up. Contour data from baseline and follow-up pullbacks were co-registered and then processed to extract several frame-wise features, e.g. areas, plaque burden, eccentricity, etc. Each pullback was divided into regions of interest (ROIs), following different criteria. Frame-wise features were condensed into region-wise markers using tools from statistics, signal processing, and information theory. Finally, a stratified 5-fold cross-validation strategy (20 repetitions) was used to train/validate an XGBoost regression models. A feature selection method before the model training was also applied. When the models were trained/validated on ROI defined by the difference between follow-up and baseline plaque burden, the average accuracy and Mathews correlation coefficient were 0.70 and 0.41 respectively. Using a ROI partition criterion based only on the baseline’s plaque burden resulted in averages of 0.60 accuracy and 0.23 Mathews correlation coefficient. An XGBoost model was capable of predicting plaque progression/regression changes in coronary vascular segments of patients treated with rosuvastatin therapy in 13 months. The proposed method, first of its kind, successfully managed to address the problem of stratification of patients at risk of coronary plaque progression, using IVUS images and standard patient clinical data.

## Introduction

Coronary artery disease (CAD) is defined by atherosclerotic plaque deposition on the arterial wall, which can trigger blood flow impairment and ultimately lead to myocardial ischemia. Predicting the regions of the artery that will suffer plaque burden increase (progression) or decrease (regression) remains an elusive major milestone in cardiology. For the past three decades, the primary tool to invasively assess atherosclerotic plaque has been intravascular ultrasound (IVUS) imaging, currently considered the gold standard for plaque quantification. A recent review^1^ showed a minor effect on plaque regression in patients with CAD treated with various therapeutic strategies that modify systemic markers such as LDL, HDL, and/or blood pressure. In such a study, the importance of IVUS imaging as a tool to guide treatment based on plaque burden and composition is highlighted under the hypothesis that plaque evolution could be predicted from such images. The IBIS-4 clinical trial^2^ is one such study, which showed that high-intensity rosuvastatin therapy over 13 months was associated with regression of coronary atherosclerosis in non-infarct-related arteries.

Such landmark studies help strengthen the body of evidence about the efficacy of drugs, create clinical guidelines, and establish consensus on standard treatments. Nevertheless, they are not suitable for a patient-specific risk prediction at the most granular scale, i.e. to discover those regions of the compromised artery that are susceptible to plaque progression or regression. Such fine-scale characterization could provide a complementary paradigm for treatment and ultimately indicate localized preventive interventions.

From now on, we distinguish scientific contributions to the field into two categories, *descriptive* and *predictive*. The former study type usually presents results of widespread classical methods from medical statistics, such as descriptive statistics and exploratory data analysis, or logistic regression models to investigate the association between plaque progression-related end-points at follow-up and baseline variables. In turn, studies in the second category, shift the focus from descriptive statistics to patient-, or artery-specific predictive models. Most of these predictive models fall into the family of machine learning models, such as support vector machines or random forests. Moreover, the outcomes delivered by these predictive models are assessed using classification metrics such as accuracy, sensitivity, specificity, and so on.

Remarkably, while the prediction of plaque evolution is a major milestone, only a small number of studies have moved from descriptive statistics to predictive models in the context of coronary artery disease. Moreover, in such studies, the model development relies on a small number of patients, limiting the models’ predictive power. Noteworthy, the inputs of the models are based on morphological and biomechanical features, by integrating image-based analysis and computational simulations to estimate hemodynamic- and mechanical-derived indexes such as wall shear stress, and plaque stress, among others.

Comprehensive reviews related to image-based computational modeling of human carotid and coronary plaque, as well as grand challenges and uncertainties in the field to develop predictive patient screening tools, can be found in^3,4^. Although several models have been proposed to capture the many factors theoretically related to plaque formation and evolution, as model complexity grows, the number of patients drastically decreases, even to the point of a single case of study. The reader is referred to^5^, for a review of the literature focused on *descriptive* and *predictive* studies on coronary plaque evolution. Most *predictive* studies incorporate heavy image processing to extract features and generate computational models of the arteries for blood flow-, wall mechanics-, species transport-, and even mass transfer simulations.

To the best of our knowledge, there is a handful of *predictive* publications, summarized in a table in the supplementary material. It is essential to point out that they all share a joint core group of authors, who proposed a computational framework that uses morphological measures from images and biomechanical indexes estimated by fluid-dynamics/mechanical simulations^6–10^.﻿ Although morphological features require simple image segmentation and processing techniques, some of them require virtual-histology-IVUS, optical coherent tomography (OCT), or even 3D quantitative coronary angiography (3DQCA) to compute the features. Moreover, on top of these more sophisticated protocols, assumptions about model parameters, and boundary conditions are also needed to perform computational simulation for calculating the biomechanical features. Regarding the end-point, always at the frame level, these studies have employed changes in lumen-, plaque-area, plaque burden, or even indexes for plaque vulnerability and risk (proposed by the same research group). In terms of classification models, they used support vector machines, random forest, discriminant analysis, and generalized linear logistical regression models, using five-fold cross-validation (at the frame level and typically without repetition), or out-of-bag for random forest methods.

In this work, we propose a predictive machine learning model to estimate the change in the percent of atheroma volume ($\Delta \hbox {PAV}$) as the end-point, using baseline lumen and vessel contours from IVUS images and patient data, e.g. age, gender, body mass index, etc. Predictions are performed at a region-wise level instead of frame-wisely. We develop a model to estimate $\Delta \hbox {PAV}$ based on an XGBoost regressor. Data is split into training/test groups, and we employ a five-fold cross-validation (at the patient level) with 20 repetitions to assess model performance. Model input consists of morphological features extracted from IVUS lumen and vessel contours. Note that the change in percent atheroma volume is the gold standard in terms of plaque quantification and assessments from the clinical point of view. The proposed model is developed with a subset of the IBIS-4 data set, spanning 140 arteries from 81 patients, becoming the largest data set ever used for plaque progression/regression prediction models.

## Materials and methods.

### Database

The reader is redirected to the seminal paper^2^ for details regarding the study design behind the IBIS-4 trial, image acquisition procedures, and population demographics. The IBIS-4 study is part of the NCT00962416 clinical trial^11^, and was published in 2014. All the patients provided written informed consent, the study was carried out in compliance with all guidelines and regulations, and the study was approved by the ethics committee and review boards of the Inselspital, University Hospital Bern (Bern, Switzerland), and all participating centers.

A 20-MHz ultrasound catheter (Eagle Eye, Volcano Cooperation, Rancho Cordova, CA) was used, at a speed of 0.5 mm/s. Images were acquired at 30 frames per second, meaning that frame spacing is 1/60 $\hbox {mm}$. Baseline (BL) and Follow-up (FU) pullbacks were acquired 13 months apart. For each (BL, FU) pullback pair, the largest common region available was assessed using dedicated software between two anatomical landmarks (e.g. distal: side branch, proximal: LM bifurcation or ostium of the RCA). Those common matching frames that were identified were used to manually identify the same anatomical region on both pullbacks. For the selected frames (mean frame spacing 0.4 mm), the lumen and the vessel contours were delineated using the same dedicated software (QIVUS, Medis, Leiden, The Netherlands).

**Figure 1:**
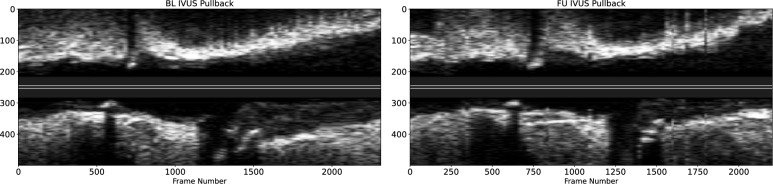
For a selected patient, longitudinal views of the baseline IVUS pullback ($\hbox {BL}$, left panel), and the corresponding follow-up IVUS pullback ($\hbox {FU}$ right panel). The horizontal axis indicates the frame number and the vertical axis indicates the pixel coordinate of the images, giving a sense of the image resolution of the IVUS frames ($480\times 480$).

A total of 140 arteries from 81 patients were included in the present study. Figure 1 presents the (BL, FU) pullback pair of IVUS images.﻿ Clinical variables definitions and demographics are provided in Table 1. The original IBIS4 study by^2^ analyzed arterial cross-sectional geometry through area-based measurements and plaque composition. The later measurements were unavailable at the time of the present study. Therefore, the set of geometric descriptors shared with the IBIS4 study is listed in Table 2.

The variable of interest in the present study, also referred to as the target variable, is the difference in the percent atheroma volume, follow-up (FU) minus baseline (BL), defined as

1 $$\begin{aligned} \Delta \hbox {PAV}= \hbox {PAV}_{\hbox {FU}} - \hbox {PAV}_{\hbox {BL}} \end{aligned}$$

We define plaque progression whenever $\Delta \hbox {PAV}>\varepsilon$ and regression whenever $\Delta \hbox {PAV}<-\varepsilon$. Condition $-\varepsilon<\Delta \hbox {PAV}<\varepsilon$ is regarded as stationary plaque. In this work, we use $\varepsilon =0$, as in the original IBIS study^2^.

**Table 1:** Baseline characteristics for the 81 patient sample.

Age, AGE	58.6 (9.9)
Male Sex, MSX(%)	75 (93)
Body Mass Index, BMI	27.5 (3.8)
Cholesterol	
High-density lipoproteins, HDL	1.1 (0.3)
Low-density lipoproteins, LDL	3.4 (0.9)
Cardiovascular risk factors (%)	
Diabetes, DIA	8 (10)
Hypertension, HYT	38 (47)
Hypercholesterolaemia$^a$, HYC	35 (43)
Current Smoker, CSK	34 (42)
Family history of CAD, FHC	24 (31)
Renal failure, RFL$^{b}$	3 (4)
Previous myocardial infarction, PMI	2 (2)
Previous PCI, PPI	1 (1)
Cardiovascular medications at 30 days (%)	
Statin use prior to enrolment, STA	7 (9)
Aspirin, ASP	81 (100)
Prasugrel, PRA	63 (78)
Clopidogrel, CLO	17 (21)
Any DAPT, DAPT	80 (99)
Beta-blocker, BB	77 (95)
ACE inhibitor, ACE	60 (74)

For continuous variables, the mean (std) is presented, for boolean variables the *n* (percentage of total) is presented. CAD: Coronary Artery Disease, PCI: Previous Percutaneous Infarction, DAPT: Dual Anti-Platelet Therapy. $^a$ Total cholesterol >5.0 mmol or 190 mg/dL or requiring treatment. $^{b}$ >60 eGFR.

**Table 2:** List of features used in the original IBIS-4 study. We refer to these features as *Clinical* features.

Clinical frame-wise features	
$\Lambda _{\hbox {L}}$	Area enclosed by lumen contour, in mm$^2$.
$\Lambda _{\hbox {V}}$	Area enclosed by vessel contour, in mm$^2$.
$\Lambda _{\hbox {P}}$	Area between vessel and lumen contours, defined as the $\Lambda _{\hbox {V}}- \Lambda _{\hbox {L}}$, in mm$^2$.
PB	Plaque burden defined as $(\Lambda _{\hbox {P}}/ \Lambda _{\hbox {V}}) \times 100$, in %.
Clinical condense features	
TLV	Volume of the lumen along a set of consecutive frames, integration of $\Lambda _{\hbox {L}}$, in mm$^3$.
TVV	Volume of the vessel a set of consecutive frames, integration of $\Lambda _{\hbox {V}}$, in mm$^3$.
TAV	Volume of the plaque along a set of consecutive frames, integration of $\Lambda _{\hbox {P}}$, in mm$^3$.
PAV	Percentage atheroma volume $(\hbox {TAV}/ \hbox {TVV}) \times 100$, in %.
Variable of interest	
$\Delta \hbox {PAV}$	The difference of the $\hbox {PAV}$ variable between follow-up ($\hbox {FU}$) and baseline ($\hbox {BL}$) at a specific region of interest, in %.

They are defined from the (lumen and vessel) area of the manually delineated contours at each frame and then condensed to volumes by numerical integration. The change in percentage atheroma volume is the variable of interest of the study.

### Frame-wise feature definition

For any given frame of a pullback, BL or FU, for which the contour delineation of the lumen (*L*) and the vessel (*V*) are available, a set of geometry-based descriptors are calculated, see Table 3. Since healthy arteries have, ideally, a circular cross-section lumen and vessel contours, with relatively uniform wall thickness, the proposed features aimed at capturing the contour geometry complexity, and deviation from such ideal references, using well-known measures such as eccentricity, circularity, and local curvatures. Also, we proposed features that aim at characterizing the plaque distribution over the contour.

**Table 3:** List of proposed geometric features based on manually defined lumen (*L*) and vessel (*V*) contours.

$\xi _{[L | V]}$	Ratio between the maximum and minimum lumen (L) or vessel (V) diameters, measured using the line that runs through the image origin and joins opposite points through the corresponding contour.
$\xi _{P}$	Distance between the centroids of the lumen and vessel contours, divided by the average between the lumen minimum and maximum diameters.
$\rho _{[L | V]}$	Percentage of circumferential angle for which the ratio between plaque thickness and lumen (*l*) or vessel (*v*) radius is over 0.2.
$\varrho$	Ratio between the maximum plaque thickness over the mean plaque thickness, along the circumferential direction.
$\psi _{[L | V]}$	Geometric definition of eccentricity for ellipses, $\sqrt{1 - (min/max)^2}$, where min and max are the smallest and larger diameters of the lumen (*L*) or vessel (*V*).
$\phi _{[L | V]}$	Geometric circularity of a polygon, $4 \pi A / P^2$, where *A* and *P* are the area and perimeter of the lumen (*L*) or vessel (*V*) contours.
$\tau _{[L | V]}$	Curvature irregularity^12^, defined as the difference between the maximum and minimum curvature of the lumen (*L*) or vessel (*V*) contour.
$\kappa _{[L | V]}$	Curvature roughness^12^, reflects the lumen surface evenness concerning the curvature, smaller values representing a more circular or even surface, and a perfect circular lumen shape will have roughness being 1. It is calculated using the following formula $\sqrt{(r/2\pi )\sum {\varkappa ^2 \Delta l}}$, where *r* is the radius of the circle best fitting the lumen or vessel contour, $\varkappa$ is local curvature, and $\Delta l$ is the local length between adjacent points).

Proposed features aimed at capturing the contour geometry complexity using well-known measures such as eccentricity and local curvatures. Also, we proposed features that aim at characterizing the plaque distribution over the contour.

### Pre-processing

**Figure 2:**
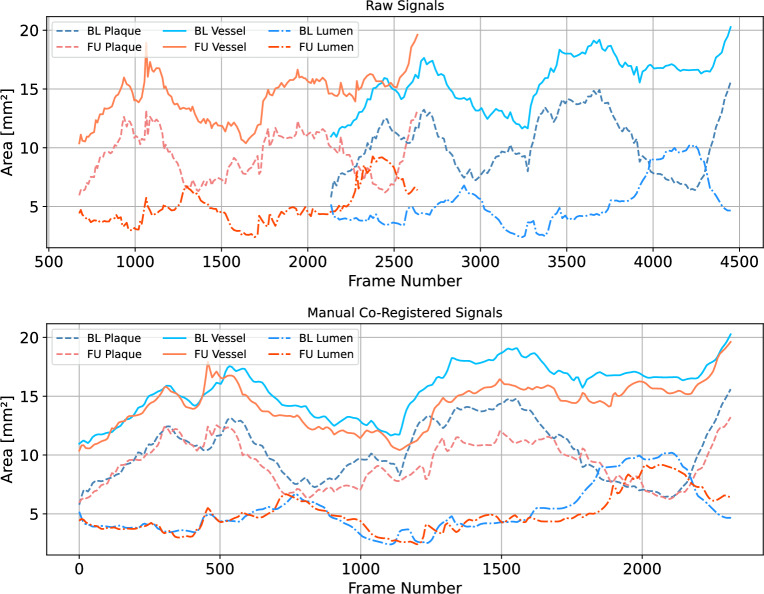
Illustration of the manual $\hbox {BL}$/$\hbox {FU}$ registration procedure for one selected pullback. Top panel: raw plaque, vessel, and lumen signals of the $\hbox {BL}$ (blue-toned) and $\hbox {FU}$ (red-toned). Bottom panel: co-registered signals. Co-registration produced anatomically consistent signals, by a two-step method: first, homogenizing frame spacing to 60 frames per second using linear interpolation; and second, manual shifting, and clipping tails of the signals until matching of the local extrema was reached..

Frame-wise features were linearly interpolated to fill gaps between unevenly spaced frames and generate longitudinal signals. Features PB and $\Lambda _{\hbox {V}}$ were used to (manually) co-register $\hbox {BL}$ and $\hbox {FU}$ signals, resulting in anatomically consistent signals, with a uniform frame spacing of 60 $\hbox {frames}/\hbox {mm}$, see Figure 2, for an illustration. Manual co-registration consisted of shifting and clipping the tails of the signals until matching of the local extrema.

For each frame-wise feature (longitudinal signals) described in Tables 2 and 3, the first derivative was computed using a central finite difference approximation. Hereafter, $\partial f$ indicates the first derivative of the frame-wise feature *f*, and it is itself a frame-wise feature represented as a longitudinal signal.

### Region of interest

A single IVUS pullback can be used to image a large portion of an epicardial coronary artery. Changes in the PB, from $\hbox {BL}$ to $\hbox {FU}$, along the entire segment can either be positive (progression) or negative (regression), in a wide range of magnitudes. Furthermore, by considering a certain threshold, the outcome could be an unchanged PB. Since the target variable, $\Delta \hbox {PAV}$, is defined over an arterial volume, it is key to define regions of interest within the pullback, for which the regression/classification model will analyze and predict the associated $\Delta \hbox {PAV}$ (value, or sign).

We defined the ROIs in 6 ways to analyze the potential and limitations of the proposed classification methodology. We propose to use (a) the complete pullback (FP criterion); (b) ROIs defined by using the difference between PB of the FU and BL (ChPAV criterion); (c) ROIs defined by using thresholding on the BL PB signal (PBR criterion), and three different fixed-length widows (W30O10, W60O20, and W120O20 criteria). Table 4 presents the criteria used for ROI definition.

**Table 4:** Definition of criteria for the generation of ROIs. We propose to assess our methodology using the complete pullback (FP), the partition using the difference between PB of the FU and BL (ChPAV), a threshold on the BL PB signal (PBR), and three different fixed-length widows (W30O10, W60O20, and W120O20).

ROI code name	Criterion
FP	Full (complete) pullback. This criterion results in larger ROIs in which sub-regions of progression or regression may occur. Nevertheless, it is useful to establish a level of comparison and also to separate sets for training and validation at a global level, as will be shown in forthcoming sections.
ChPAV	Each pullback is divided into ROIs where the difference between $\hbox {BL}$ and $\hbox {FU}$ of PB is positive (or negative) in all (interpolated) frames of the ROI. Note that this approach for ROI definition is not applicable in clinical scenarios, where a new pullback is being analyzed and the $\hbox {FU}$ data is not available. Nevertheless, for studying the potential of the method, this methodology provides an ideal ROI definition for $\Delta \hbox {PAV}$ regression/classification models. Regions with $|{\text{PB}}_{{{\text{FU}}}} - {\text{PB}}_{{{\text{BL}}}} | < 0.5\%$ were discarded. Regions spanning less than 15 interpolated frames, i.e. less than 0.25 mm, were discarded.
PBR	The $\hbox {BL}$ PB is binarized into three categories, ${\text {PB}}\in [0,30)$, ${\text {PB}}\in [30,60)$, and ${\text {PB}}\in [60,100]$. Then, continuous frames with the same category are packed into ROIs. Regions spanning less than 15 interpolated frames, i.e. less than 0.25 mm, were discarded.
W30O10	ROIs are divided using a fixed window size of 30 (interpolated) frames, which is shifted at 30 frames over the complete pullback. The ROI generation is repeated using 0, 10, 20 frames as offsets. Given the frames-spacing, these ROIs span 0.5 mm in length.
W60O20	ROIs are divided using a fixed window size of 60 (interpolated) frames, which is shifted at 60 frames over the complete pullback. The ROI generation is repeated using 0, 20, 40 frames as offsets. Given the frames-spacing, these ROIs span 1.0 mm in length.
W120O20	ROIs are divided using a fixed window size of 120 (interpolated) frames, which is shifted at 120 frames over the complete pullback. The ROI generation is repeated using 0, 20, 40, 60, 80, 100 frames as offsets. Given the frame-spacing, these ROIs span 2.0 mm in length.

### Condensed feature definition

All the frame-wise features presented here were condensed into ROI-wise features using classical statistical indexes such as the median, Shannon’s entropy (H), and discrete Fourier Transform (FFT). Moreover, the ROI’s length is also used as a condensed feature. Each of these ROI-wise features is a real number, and a set of *n* features represents each ROI as a point in an $\mathbb {R}^n$ space. The arterial label, eg. LAD, LCx, or RCA, is the only categorical variable used in this work. Table 5, presents all ROI-wise features.

**Table 5:** List of condensed features defined within each ROI. Frame-wise features are condensed into real-number features using classical statistical indexes such as the median, using the Shannon entropy (H), and discrete Fourier Transform (FFT). In addition, the arterial label and ROI’s length are also used. as condensed features..

Statistics over each frame-wise feature (*f*)	
$\hbox {MED}(f)$	The median of *f* in the ROI.
$\hbox {IQR}(f)$	The interquartile range of *f* in the ROI, i.e. difference between percentiles 75% - 25%.
$\hbox {ICR}(f)$	The central 80% range of *f* in the ROI, i.e. difference between percentiles 90% - 10%.
Information-theory over each frame-wise feature (*f*)	
$\hbox {H}(f)$	Shannon’s Entropy from an approximation of the discrete probability function of the frame-wise feature *f*.
Fourier analysis over frame-wise feature (*f*)	
$\hbox {FFT}^m(f)$	The magnitude of the 1st Fourier harmonic, of the point-wise feature *f*.
$\hbox {FFT}^p(f)$	The phase of the 1st Fourier harmonic, of the point-wise feature *f*.
Macro features	
$\mathscr {A}$	The arterial label, e.g. LAD, LCx, RCA, etc, that was interrogated.
$\ell$	The length of the ROI.

### Machine learning model

We use an XGBoost regressor model^13^. Hyperparameters were defined empirically and remained fixed for all the tests performed in this work. The XGBoost setup consists of 256 estimators with a maximum depth of 12 levels, and the loss function was the squared error. The complete list of hyperparameters is given in the Appendix.

### Feature handling

We use an initial feature set, followed by standardization, and feature selection. Regarding the initial feature set, we employ the following notation for the three alternatives studied here.

- $\mathscr {S}^a$: set of clinical features, see Table 1;
- $\mathscr {S}^b$: set $\mathscr {S}^a$ plus set of geometric features from the original IBIS-4 study, see Table 2;
- $\mathscr {S}^d$: set $\mathscr {S}^b$ plus all condensed features presented in Table 5;

For a given feature *d*, we standardize it (feature is now called $\hat{d}$), simply by computing the mean ($\bar{d}$) and standard deviation ($\bar{\bar{d}}$), and apply the following transformation $\hat{d} = (d - \bar{d})/\bar{\bar{d}}$.

Finally, we use feature selection of the k-best features using mutual information as a proxy classification function. See the Appendix for implementation details. Following the feature set notation defined here, this results in the following feature sets:

- $\mathscr {S}^d_{k=8}$: set $\mathscr {S}^d$, where we select the best $k=8$ features (see also the Appendix).
- $\mathscr {S}^d_{k=32}$: set $\mathscr {S}^d$, where we select the best $k=32$ features (see also the Appendix).

### Training/test methodology

A repeated stratified k-fold cross-validation (RSKFCV) strategy was used for training and testing. Folds were always defined at the pullback level, i.e. using the FP criterion for ROI definition. Then, depending on the experiment, the corresponding ROIs for each pullback were accordingly assigned to the training or testing sets in the fold. Moreover, fixing the random generator’s seed parameter of the RSKFCV implementation ensures that all experiments are trained/tested over the same pullback sets, what is changed are the ROIs defined inside the pullbacks. Stratification was based on the sign of the $\Delta \hbox {PAV}$ variable, at the pullback level. The following pipeline is performed for each model/scenario.

1. Choose the initial feature set from the ones defined in Sect. "Feature handling"
2. Perform feature standardization using the training samples to compute standardization parameters, see Sect. "Feature handling".
3. Feature selection, see Sect. "Feature handling".
4. Train an XGBoost regressor model configured as detailed in Sect. "Machine learning model".
5. Perform prediction over the test set. This involves standardization, feature selection (both steps using parameters from the training set), and then forward-passing through the model.
6. Compute prediction metrics over the test set: mean absolute error (MAE), mean square error (MSE), Pearson’s correlation coefficient (*r*).
7. Perform classification over the regression estimation using 0 as the threshold to classify in estimated progression or regression.
8. Compute prediction metrics over the test set. Accuracy (ACC), Mathews Correlation Coefficient (MCC), F1-score for $\Delta \hbox {PAV}<0$ class ($\hbox {F1}^-$), F1-score for $\Delta \hbox {PAV}>0$ class ($\hbox {F1}^+$), and the average F1-score between the two classes ($\hbox {F1}^a$).

**Figure 3:**
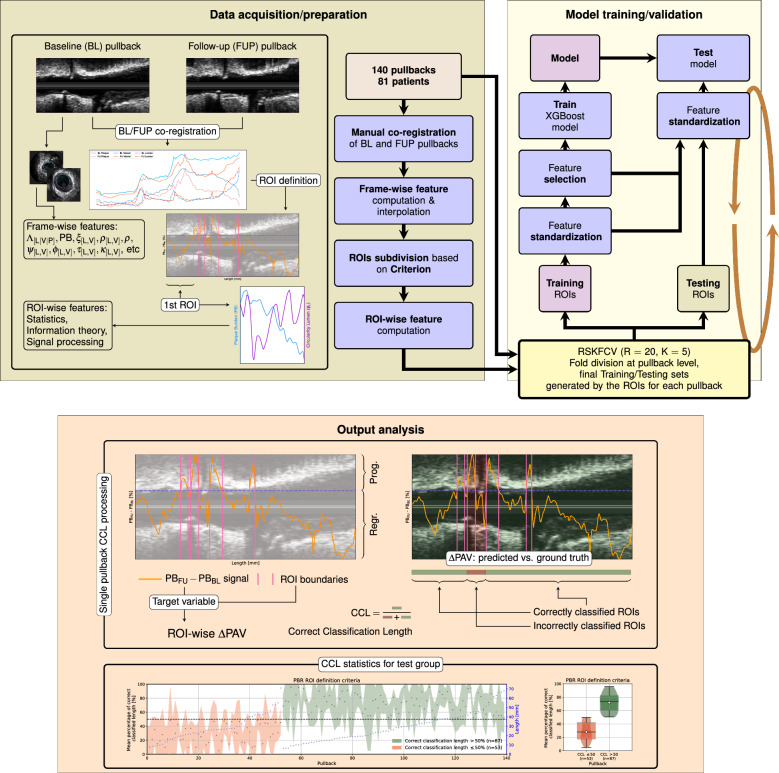
Illustration of the complete processing pipeline. The data preparation block comprises co-registration between BL and FU pullbacks, followed by signal interpolation, frame-wise feature computation, ROI definition, and ROI-wise feature characterization using elements from statistics, information theory, and signal processing. The cross-validation folds are defined at the pullback level, but actual partitions are defined from corresponding ROIs. Training samples are standardized, and a feature extraction algorithm is used (optionally) to reduce the dimension of the feature space. An XGBoost regressor is trained and then used to predict $\Delta \hbox {PAV}$ data for the current fold. Finally, ROI-wise comparison of the predicted values by the regressor, and the resulting classification into progression/regression is performed, and several performance metrics are computed and analyzed.

After iterations are completed, the mean and standard deviation of each prediction metric are gathered for assessing and comparing the performance. Figure 3 illustrates the complete pipeline to perform one regression/classification experiment.

All tests reported in this study were performed using the same data set partitions at pullback level, for the RSKFCV loop. Also, the XGBoost configuration remained fixed for all tests. Regarding the feature selection, it also remained fixed (whenever used), the only parameter that changed was the number of features to be selected (either 8 or 32). The reader is directed to the Appendix for more details.

We make use of Shapley additive explanations (SHAP) values, proposed by^14^, to measure the impact of each feature on the output of the XGBoost model. The SHAP value of a feature can be computed for each sample and then averaged over the test set. The larger the mean absolute SHAP value of a feature over the test set, the greater the impact on the prediction of the model relative to the mean prediction over the test set.

## Results

Table 6 presents basic statistics for the $\Delta \hbox {PAV}$ variable considering the different criteria to define the ROI partitions. Furthermore, the total number of samples per class (progression ”$+$” or regression ”−”) is reported, as well as the distribution in terms of mean, (std), [min, max] of the number of samples per class for training (tr) and test (te) sets for the RSKFCV iterations. Observe that the class representing plaque progression ($+$) is the minority class for all partitions. Moreover, the class imbalance is more notorious at FP partition (34%), and it is most balanced for the ChPAV partition (47%), while the rest of the partitioning criteria exhibit a similar prevalence of the progression class (between 40% and 43%). In terms of sample size, the FP partition contains the smallest number of samples ($n=140$), followed by PBR with ($n=763$) which is almost 3 times smaller than the ChPAV partition sample size ($n=2167$). The other partitions are much larger in terms of sample size.

The statistics of the prediction metrics for all tests included in this work are presented in Table 7. These tests are characterized by the dataset (ROI definition criterion), and by the set of features/feature-selection-strategy employed.

For an in-depth analysis, we considered the ChPAV and PBR ROI definition criterion. The former is useful as a reference because it represents the best possible ROI partition criterion since all frames in a given partition will render either progression or regression. In turn, the PBR criterion was chosen because it rendered the best results among the rest of the ROI partition criteria. Moreover, we selected the best-performing feature set according to the mean MCC metric, as presented in Table 7. Specifically, the $\mathscr {S}^d_{k=32}$ feature set for both, the ChPAV and PBR criteria, is used for an in-depth analysis.

**Figure 4:**
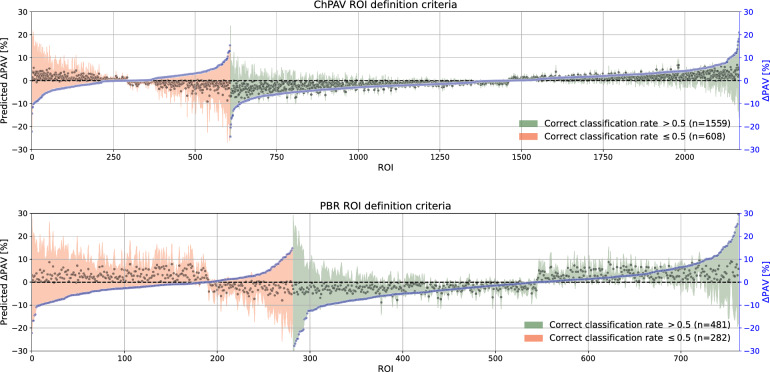
Distribution of mean $\Delta \hbox {PAV}$ prediction and absolute error segregated as a function of the true $\Delta \hbox {PAV}$. Mean prediction per ROI as is shown with black markers (left axis), the colored areas are computed by adding or subtracting to each black marker the mean absolute error of the ROI (left axis). Samples are separated by the correct classification rate (red: CCR$\le 0.5$, green: CCR$>0.5$), and ordered by the sample’s $\Delta \hbox {PAV}$ value, blue markers (right axis). Top panel corresponds to the ChPAV ROI definition criterion, and the bottom panel plot corresponds to the PBR definition criterion..

Next, we exploited the fact that each ROI was used in 20 different models as part of the test set during the RSKFCV, therefore the means of the prediction and absolute error for each ROI are computed for the 20 models, which differ among ROIs. The correct classification rate (CCR) represents the number of times a given ROI was classified correctly by the set of 20 models. This metric is used to discriminate between CCR$>0.5$ and those with CCR$\le 0.5$, i.e. those ROIs that were more often classified correctly than incorrectly. In Fig. 4, ROIs were divided by their CCR status and ordered by the true $\Delta \hbox {PAV}$ value (blue marker). The top panel corresponds to the ChPAV criterion and the bottom panel to the PBR criterion. Reddish regions stand for mostly incorrectly classified ROIs (CCR$\le 0.5$), and greenish regions for mostly correctly classified ROIs (CCR$>0.5$). Black markers represent the mean prediction per ROI (as given by the 20 models), and the envelope-colored area around those markers is obtained by adding and subtracting the mean absolute error. This allows both a qualitative and quantitative comparison of the mean accuracy of the models. Note that the mean absolute error (reddish and greenish regions) seems to be related to the magnitude of the $\Delta \hbox {PAV}$, except for the ROIs when CCR$\le 0.5$ in the PBR criterion when $\Delta \hbox {PAV}<0$ in the prediction. Another qualitative conclusion that can be extracted from the plots is that the mean prediction seems to be limited in a more narrow range than the actual $\Delta \hbox {PAV}$, more observations on this are made in the following paragraph.

**Figure 5:**
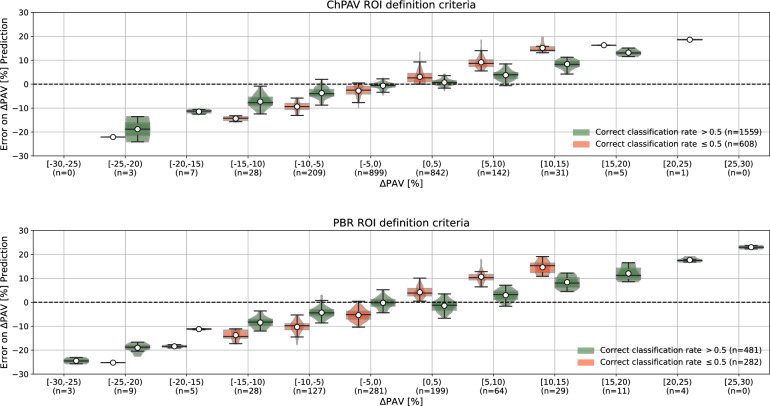
Violin-/box-plots of the mean $\Delta \hbox {PAV}$ prediction per ROI for each range of the true $\Delta \hbox {PAV}$. Mean error in the prediction of $\Delta \hbox {PAV}$ for the different ROIs, segregated according to the correct classification rate (red: CCR$\le 0.5$, green: CCR$>0.5$), and ordered from left to right according to the $\Delta \hbox {PAV}$ value range. The top panel corresponds to the ChPAV ROI definition criterion, and the bottom panel plot corresponds to the PBR definition criterion..

In Fig. 5, besides separating ROIs by their CCR status, we discretized them according to their $$\Delta \hbox {PAV}$$ in even intervals. Finally, we use the mean error of each ROI over the 20 models in which it belongs to the test group, to construct a box/violin-plot. It is noteworthy that distribution on both cases, ChPAV and PBR, condense 96% and 88% on the $$[-10,10)$$ $$\Delta \hbox {PAV}$$ interval respectively. This interval corresponds to the prediction range mentioned in the previous paragraph. Moreover, classification rates outside of the interval are mostly correct. Which somehow shows that the prediction seems robust to out-layers.

**Figure 6:**
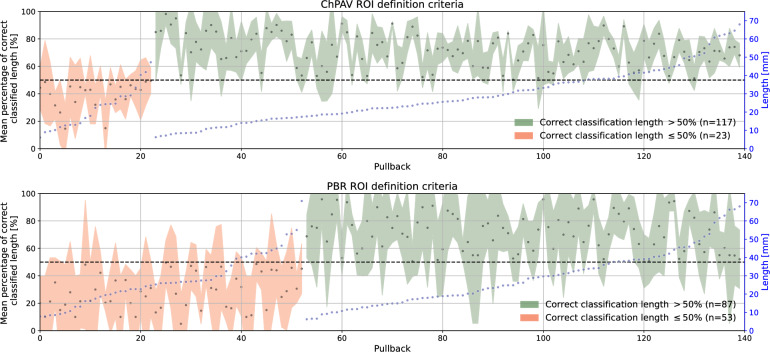
For each pullback, the CCL is computed and used to group samples. The length per pullback (blue marker, right axis), is used to sort them. Black markers (right axis) represent the CCL, that is, the average length of a pullback that was correctly classified (left axis). The colored areas are created by adding and subtracting the std of CCL as predicted by the 20 models. Top panel plots samples from the ChPAV ROI definition criterion, and the bottom panel plots samples for the PBR criteria.

**Figure 7:**
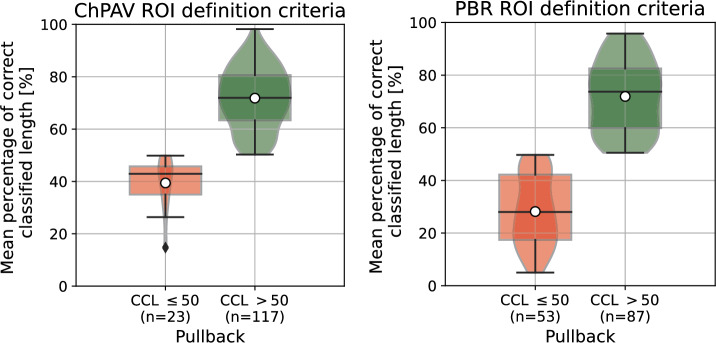
Box-/Violin-plots for the mean percentage of correct classified lengths per pullback grouped by CCL>0.5. Left panel plots samples from the ChPAV ROI definition criterion, and the right panel plots samples for the PBR criteria.

In Fig. 6, the concept of mean percentage of correct classified length (CCL) per pullback is introduced, and it is used to dichotomize pullbacks by a threshold of CCL$>0.5$. Recalling the cross-validation strategy explained in Sect. "Training/test methodology", folds are defined at pullback level, which ensures that all ROIs of a given pullback are used for training xor testing. Therefore, again we use for each pullback only the 20 models in which it was used as a test sample, to compute the mean and std of the CCL. Specifically, the CCL index for a given pullback is computed as the ratio of the summation of the lengths of ROIs correctly classified and the summation of all its ROIs lengths. In qualitative terms, there is no association of the CCL to the length of the pullback. The distributions of CCL are presented in the box-/violin-plots in Fig. 7. It can be noted that for CCL$>50$%, the mean and median are close to 70%, with an interquartile range (IQR) of around 20%, being slightly larger for the PBR case compared to the ChPAV one, also the distribution looks more homogeneous in the PBR case. Regarding the cases for CCL$\le 50$%, the ChPAV case presents half the number of samples in this category than the PBR case. Moreover, the distribution yields mean and median values close to 40% and an IQR around 10%. While the PBR case features more spread distributions of CCL, with mean and median close to 30% and an IQR around 25%.

**Figure 8:**
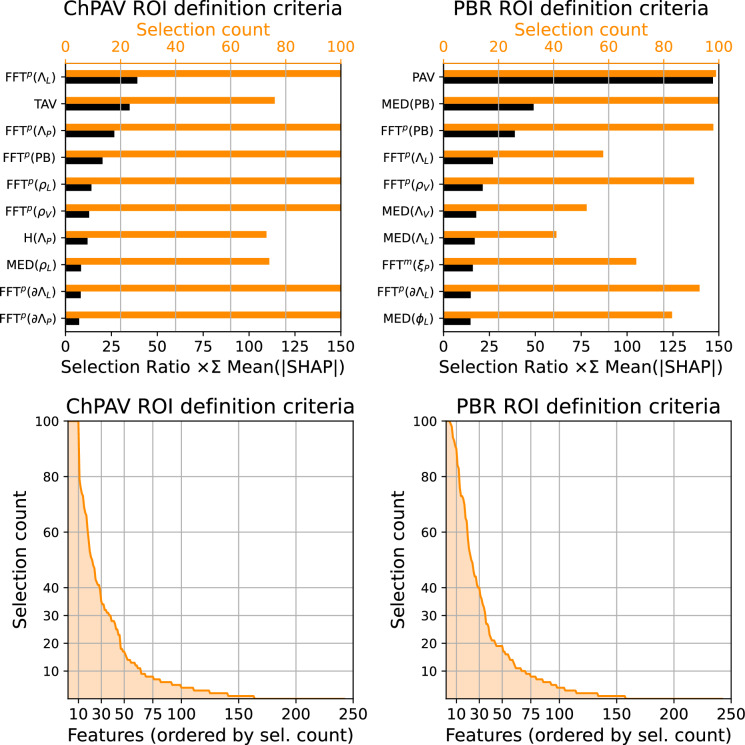
The top panel presents the selection count and the weighted total mean absolute SHAP values of the top ten features for the ChPAV (left) and PB (right) ROI definition criterion. A sorted histogram of the selection count is presented in the bottom panel, again for the ChPAV (left) and PB (right) criterion.

Regarding the initial set of features, feature selection, and feature impact on model prediction, we present the following analysis. Scenarios presented here, namely ChPAV and PBR, used the same initial feature set and selection strategy, $\mathscr {S}^d_{k=32}$. The top panel in Fig. 8 presents the selection count and the selection ratio times the summation of the mean absolute SHAP value. Note that the mean absolute SHAP value of a feature is computed for each of the 100 models used during the RSKFCV procedure, using the corresponding test samples. Since feature selection of k=32 is performed prior to model training, we count the number of times a feature was selected throughout the 100 models and then compute the selection ratio as the selection count over 100, and we use it to weight the mean absolute SHAP value of the feature. Ordering by this weighted value and retrieving the top ten, we obtain the features with more impact on model predictions over the 100 models. The mid panel of Fig. 8 presents a histogram ordered by the selection count. This visualization shows that from the initial set of 242 features, the selection process always selects the same  10 (selection count equals 100), and that this count rapidly drops around 20 for the  50th feature. From there different features are rarely selected, as seen in the selection count plot.

Table 8 presents the selection count and total mean absolute SHAP values times the selection ratio (SR) values for all clinical features defined in Tables 1 and 2, in addition to the arterial label ($\mathscr {A}$) and ROI length ($\ell$). Interestingly, the $\hbox {TLV}$, $\hbox {TVV}$, and $\ell$ were rarely selected and when selected the impact in the models in terms of SHAP values was low, for the ChPAV and the PBR cases. Instead, the $\hbox {TAV}$ impacted the ChPAV models considerably, but its contribution to the PBR models was poor. Overall, the $\hbox {PAV}$ was the most impactful feature regarding the PBR models, while in the ChPAV case, the $\hbox {PAV}$ contributed weakly. Also, although the arterial label $\mathscr {A}$ reached a high selection count, its impact was weak on the PBR models and was close to zero on the ChPAV case. In terms of the clinical variables defined at the patient level, they were seldom selected within the ChPAV cases, and, consequently, the overall impact was close to zero. In turn, for the PBR case, the MSX, DAPT, and HDLwere selected in all 100 models, although their overall global impact was weak. As for the rest of the clinical features, although they were selected more often in the PBR than in the ChPAV case, displayed poor relevance.

**Figure 9:**
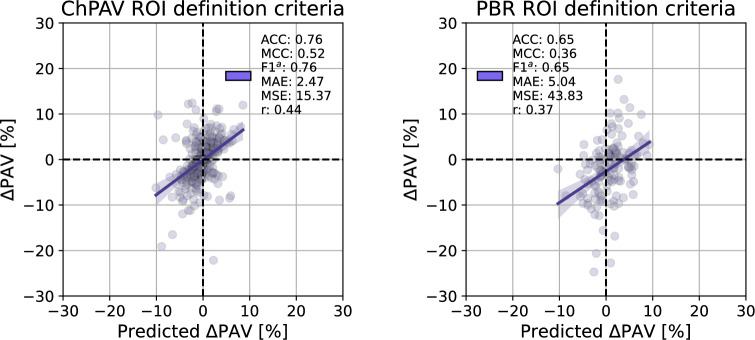
Model prediction vs $\Delta \hbox {PAV}$ for the models with the largest MCC for the ChPAV (left) and PBR (right) ROI definition criteria.

**Figure 10:**
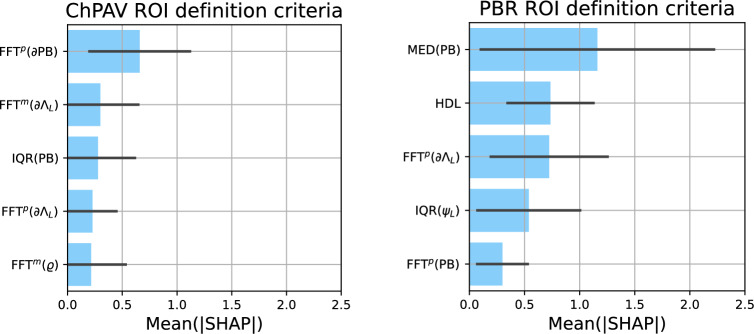
Mean absolute SHAP values (blue bars), with corresponding standard deviations (black lines), for the top five features with more impact on model predictions. The models with the largest MCC for the ChPAV case (left) and for the PBR case (right) were selected.

**Figure 11:**
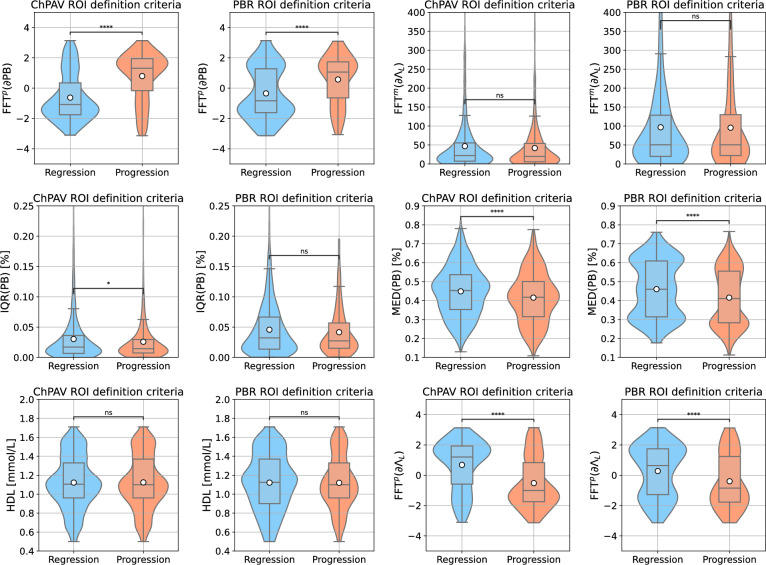
Violin-/box-plots of the top 3 features with more impact on the best-performing models in the scenarios ChPAV and PBR regarding the ROI definition criterion.

We now focus the analysis on the models that resulted in the best prediction performance according to the MCC metric. Again, for the ChPAV and PBR scenarios using $\mathscr {S}^d_{k=32}$ feature management setup. Figure 9 presents the regression plots and their prediction metrics. Importantly, the ChPAV scenario renders better performance because ROIs are built, by definition, using the regression/progression feature as a proxy. Using this scenario as a reference, the PBR case resulted in almost double the mean absolute error (MAE=5.04%), moderate linear, and Mathew’s correlation ($r=0.37$ and MCC=0.36) which are 0.07 and 0.16 lower than the ChPAV scenario. In terms of accuracy and mean F1-scores, we get fairly good results (ACC$=\hbox {F1}^a=0.65$), compared to 0.76 in the ChPAV case. Regarding the most influential features for these scenarios, Fig. 10 shows the mean absolute SHAP values of the five most influential features. The top three features of each model were selected to visualize the entire ROI sample distributions in both scenarios, ChPAV and PBR, see Fig. 11. The foremost features are those related to plaque burden (PB) and lumen-area ($\Lambda _{\hbox {L}}$), according to mean absolute SHAP values. Namely, the $\hbox {FFT}^p(\partial {\text {PB}})$, $\hbox {FFT}^m(\partial \Lambda _{\hbox {L}})$, and $\hbox {IQR}( {\text {PB}})$ for the ChPAV case and the $\hbox {MED}( {\text {PB}})$, and $\hbox {FFT}^p(\partial \Lambda _{\hbox {L}})$for the PBR case. Note that in the PBR case, the clinical feature HDL is the second most relevant feature for the model according to the mean absolute SHAP values. Nevertheless, as can be seen in Fig. 11, the mean HDL is not different between the regression/progression groups when taken as ROI level for ChPAV or PBR. This can be explained because HDL is defined at the patient level, meaning that for all ROIs of a pullback (regardless of the $\Delta \hbox {PAV}$ sign), the HDL is the same. Therefore, it is the interaction of HDL with the other features that produce an impact on the model output. Analogous reasoning can be used to explain the impact of other patient-level clinical features on this model and the impact of other features for which no statistically significant difference in mean values among the regression/progression groups is observed.

**Table 6:** For each ROI partition criterion, we present the sample Mean (std) of $\Delta \hbox {PAV}$ and the total number of ROIs (*n*) and the number of ROIs of class regression ($n^-$) and class progression ($n^+$).

ROI	$\Delta \hbox {PAV}$	*n*	$n^-$	$n^+$	$n^-_{\hbox {tr}}$	$n^+_{\hbox {tr}}$	$n^-_{\hbox {te}}$	$n^+_{\hbox {te}}$
FP	− 1.01 (3.47)	140	92	48	73.6 (0.49) [73,74]	38.4 (0.49) [38,39]	18.4 (0.49) [18,19]	9.6 (0.49) [9,10]
ChPAV	− 1.31 (8.35)	2167	1146	1021	916.8 (20.79) [861,961]	816.8 (19.32) [762,854]	229.2 (20.79) [185,285]	204.2 (19.32) [167,259]
PBR	− 1.02 (6.90)	763	454	309	363.2 (11.58) [334,391]	247.2 (9.87) [223,269]	90.8 (11.58) [63,120]	61.8 (9.87) [40,86]
W30O10	− 0.91 (6.94)	23821	13656	10165	10924.8 (233.75) [10372,11435]	8132 (196.57) [7695,8669]	2731.2 (233.75) [2221,3284]	2033 (196.57) [1496,2470]
W60O20	− 0.91 (6.70)	11734	6734	5000	5387.2 (118.24) [5099,5646]	4000 (98.4) [3786,4282]	1346.8 (118.24) [1088,1635]	1000 (98.4) [718,1214]
W120O20	− 0.90 (6.26)	11314	6580	4734	5264 (122.21) [4946,5530]	3787.2 (97.11) [3574,4088]	1316 (122.21) [1050,1634]	946.8 (97.11) [646,1160]

The right part of the table provides the statistics (mean, std, minimum, and maximum) for the number of ROIs in the classes regression and progression for the training set $(\cdot )_{\hbox {tr}}$ and for the testing set $(\cdot )_{\hbox {te}}$ in the RSKFCV process.

**Table 7:** Predictive capabilities of the model trained/tested under different ROI definition criteria using the same RSKFCV procedure, with 20 repetitions and 5 folds. For each metric, the mean (std) and [min, max] values are reported.

ROI	F. Set	$\hbox {ACC}$	$\hbox {MCC}$	$\hbox {F1}^a$	$\hbox {F1}^-$	$\hbox {F1}^+$	MAE	MSE	*r*
FP	$\mathscr {S}^a$	*0.65 (0.08) [0.5,0.82]*	*0.21 (0.17) [-0.22,0.62]*	*0.6 (0.09) [0.39,0.81]*	*0.73 (0.07) [0.46,0.87]*	0.46 (0.13) [0.12,0.76]	*2.81 (0.35) [1.99,3.52]*	*13.38 (3.31) [6.25,22.24]*	*0.35 (0.15) [-0.11,0.67]*
	$\mathscr {S}^b$	0.43 (0.11) [0.21,0.71]	0.03 (0.18) [-0.5,0.47]	0.39 (0.12) [0.18,0.7]	0.30 (0.23) [0.00,0.76]	*0.48 (0.09) [0.17,0.67]*	3.62 (1.06) [1.87,7.46]	21.19 (11.15) [5.6,69.21]	0.05 (0.22) [-0.43,0.52]
	$\mathscr {S}^d$	0.55 (0.1) [0.29,0.82]	-0.02 (0.19) [-0.51,0.6]	0.48 (0.1) [0.24,0.8]	0.66 (0.1) [0.33,0.86]	0.31 (0.14) [0.00,0.74]	2.97 (0.45) [1.9,4.23]	15.66 (5.05) [5.2,32.76]	-0.06 (0.21) [-0.52,0.41]
	$\mathscr {S}^d_{k=8}$	0.54 (0.09) [0.25,0.71]	-0.02 (0.17) [-0.47,0.35]	0.48 (0.09) [0.2,0.67]	0.63 (0.14) [0.00,0.80]	0.33 (0.14) [0.00,0.62]	3.09 (0.49) [1.93,4.48]	16.08 (4.88) [6.63,31.99]	0.01 (0.2) [-0.53,0.43]
	$\mathscr {S}^d_{k=32}$	0.56 (0.1) [0.36,0.79]	0.01 (0.21) [-0.47,0.56]	0.5 (0.1) [0.26,0.78]	0.67 (0.09) [0.47,0.86]	0.33 (0.15) [0,0.73]	2.94 (0.46) [2.01,4.09]	15.05 (4.58) [6.02,26.69]	0.03 (0.2) [-0.38,0.51]
ChPAV	$\mathscr {S}^b$	0.51 (0.02) [0.46,0.56]	0.03 (0.04) [-0.07,0.13]	0.51 (0.02) [0.46,0.56]	0.53 (0.05) [0.38,0.64]	0.49 (0.04) [0.32,0.58]	3.58 (0.28) [3.01,4.38]	23.69 (3.55) [16.58,33.87]	0.05 (0.05) [-0.1,0.17]
	$\mathscr {S}^d$	***0.70 (0.02) [0.64,0.75]***	0.40 (0.04) [0.29,0.49]	***0.70 (0.02) [0.64,0.74]***	***0.72 (0.02) [0.66,0.77]***	***0.68 (0.02) [0.63,0.73]***	***2.57 (0.19) [2.01,3.08]***	***15.86 (2.52) [9.51,22.84]***	***0.42 (0.06) [0.28,0.56]***
	$\mathscr {S}^d_{k=8}$	0.67 (0.02) [0.63,0.73]	0.35 (0.04) [0.26,0.46]	0.67 (0.02) [0.63,0.73]	0.70 (0.02) [0.65,0.74]	0.65 (0.03) [0.58,0.72]	2.97 (0.18) [2.4,3.38]	17.52 (2.19) [11.46,22.84]	0.35 (0.04) [0.23,0.47]
	$\mathscr {S}^d_{k=32}$	***0.70 (0.02) [0.66,0.76]***	***0.41 (0.04) [0.32,0.52]***	***0.70 (0.02) [0.66,0.76]***	***0.72 (0.02) [0.67,0.79]***	***0.68 (0.02) [0.63,0.75]***	2.6 (0.2) [2.06,3.1]	16 (2.52) [10.02,21.57]	***0.42 (0.06) [0.24,0.56]***
PBR	$\mathscr {S}^b$	0.50 (0.07) [0.36,0.68]	0.10 (0.09) [-0.13,0.34]	0.47 (0.09) [0.27,0.66]	0.39 (0.19) [0.02,0.74]	0.55 (0.05) [0.38,0.67]	5.86 (0.91) [3.93,9]	60.28 (15.15) [26.81,108.82]	0.16 (0.12) [-0.14,0.44]
	$\mathscr {S}^d$	*0.60 (0.04) [0.46,0.69]*	0.19 (0.08) [-0.06,0.37]	*0.59 (0.04) [0.45,0.68]*	*0.65 (0.05) [0.49,0.75]*	0.53 (0.06) [0.33,0.67]	*5.03 (0.52) [4.06,6.2]*	*48.21 (10.96) [28.54,73.43]*	0.27 (0.07) [0.07,0.44]
	$\mathscr {S}^d_{k=8}$	0.58 (0.05) [0.41,0.69]	0.19 (0.09) [-0.02,0.39]	0.57 (0.05) [0.39,0.68]	0.58 (0.1) [0.21,0.74]	0.55 (0.08) [0.22,0.67]	5.91 (0.84) [4.32,8.1]	60.06 (15.03) [32.46,107.02]	0.26 (0.07) [0.11,0.4]
	$\mathscr {S}^d_{k=32}$	*0.60 (0.05) [0.42,0.68]*	*0.23 (0.08) [0.02,0.36]*	*0.59 (0.05) [0.38,0.68]*	0.62 (0.08) [0.20,0.75]	*0.57 (0.05) [0.35,0.67]*	5.42 (0.71) [4.09,8.1]	52.24 (13.12) [30.54,105.19]	*0.30 (0.08) [0.00,0.5]*
W30O10	$\mathscr {S}^b$	0.52 (0.03) [0.45,0.61]	0.07 (0.05) [-0.08,0.21]	0.51 (0.03) [0.4,0.6]	0.5 (0.09) [0.22,0.67]	*0.52 (0.06) [0.36,0.61]*	7.05 (0.75) [5.59,8.97]	84.25 (17.72) [50.02,127.72]	0.09 (0.08) [-0.09,0.27]
	$\mathscr {S}^d$	0.49 (0.06) [0.38,0.61]	0.04 (0.07) [-0.18,0.23]	0.47 (0.06) [0.35,0.61]	0.41 (0.16) [0.16,0.72]	*0.52 (0.07) [0.3,0.61]*	6.63 (0.81) [4.91,8.71]	74.9 (16.92) [41.85,127.09]	0.06 (0.09) [-0.15,0.33]
	$\mathscr {S}^d_{k=8}$	0.55 (0.03) [0.43,0.63]	0.08 (0.05) [-0.03,0.21]	*0.53 (0.04) [0.32,0.6]*	0.59 (0.1) [0.05,0.72]	0.46 (0.07) [0.15,0.59]	*5.83 (0.74) [4.82,10.05]*	*59.33 (14.92) [39.42,154.96]*	0.12 (0.07) [-0.03,0.3]
	$\mathscr {S}^d_{k=32}$	*0.57 (0.03) [0.5,0.64]*	*0.1 (0.04) [-0.02,0.19]*	*0.53 (0.03) [0.45,0.59]*	*0.66 (0.05) [0.47,0.75]*	0.39 (0.09) [0.19,0.56]	6.04 (0.69) [4.97,8.04]	63.33 (13.67) [43.49,110.01]	*0.16 (0.05) [0.01,0.28]*
W60O20	$\mathscr {S}^b$	0.49 (0.04) [0.37,0.61]	0.04 (0.07) [-0.17,0.22]	0.47 (0.05) [0.35,0.6]	0.41 (0.12) [0.13,0.69]	*0.53 (0.04) [0.33,0.62]*	6.81 (0.87) [4.62,9.41]	81.11 (21.24) [40.48,155.94]	0.04 (0.1) [-0.24,0.31]
	$\mathscr {S}^d$	0.49 (0.05) [0.39,0.61]	0.03 (0.07) [-0.19,0.21]	0.46 (0.06) [0.31,0.58]	0.41 (0.17) [0.05,0.7]	0.51 (0.08) [0.26,0.61]	6.14 (0.72) [4.06,7.93]	65.25 (13.51) [30.57,106.85]	0.04 (0.08) [-0.15,0.26]
	$\mathscr {S}^d_{k=8}$	0.52 (0.04) [0.39,0.62]	0.07 (0.06) [-0.12,0.22]	0.51 (0.06) [0.3,0.61]	0.5 (0.14) [0.01,0.7]	0.52 (0.06) [0.3,0.62]	5.89 (0.77) [4.64,8.98]	58.91 (13.65) [36.81,114.38]	0.13 (0.08) [-0.05,0.32]
	$\mathscr {S}^d_{k=32}$	*0.56 (0.03) [0.47,0.64]*	*0.1 (0.05) [-0.01,0.23]*	*0.54 (0.03) [0.45,0.61]*	*0.61 (0.08) [0.32,0.74]*	0.47 (0.07) [0.27,0.59]	*5.38 (0.5) [4.5,6.76]*	*51.59 (8.9) [35.36,78.84]*	*0.15 (0.06) [0.02,0.31]*
W120O20	$\mathscr {S}^b$	0.49 (0.04) [0.39,0.66]	0.04 (0.07) [-0.12,0.2]	0.48 (0.05) [0.36,0.56]	0.44 (0.11) [0.19,0.77]	*0.52 (0.06) [0.31,0.64]*	6.49 (0.78) [4.75,8.54]	73.82 (18.29) [42.52,130.85]	0.04 (0.1) [-0.21,0.34]
	$\mathscr {S}^d$	0.47 (0.05) [0.4,0.61]	0.02 (0.06) [-0.16,0.2]	0.45 (0.05) [0.35,0.59]	0.38 (0.14) [0.14,0.69]	*0.52 (0.06) [0.3,0.61]*	5.72 (0.6) [3.92,7.61]	56.63 (10.46) [28.25,92.32]	0.03 (0.07) [-0.16,0.21]
	$\mathscr {S}^d_{k=8}$	0.49 (0.04) [0.39,0.59]	0.03 (0.05) [-0.07,0.16]	0.46 (0.07) [0.28,0.57]	0.41 (0.19) [0,0.68]	0.51 (0.08) [0.29,0.63]	5.73 (1.08) [4.28,8.95]	55.88 (17.8) [32.15,112.97]	0.07 (0.08) [-0.09,0.27]
	$\mathscr {S}^d_{k=32}$	*0.54 (0.04) [0.39,0.62]*	*0.09 (0.05) [-0.02,0.21]*	*0.52 (0.04) [0.36,0.6]*	*0.55 (0.11) [0.18,0.72]*	0.5 (0.06) [0.32,0.6]	*5.11 (0.56) [4.08,6.6]*	*45.73 (9.01) [27.63,70.72]*	*0.15 (0.07) [-0.03,0.31]*

The global maximum mean values per column are highlighted in bold and italic font, while the local maximum values, i.e. per ROI partition criterion, are highlighted in italic font. The columns headers stand for: method for the definition of the Region of Interest (ROI); Feature set (F. Set); Accuracy (ACC); Mathew correlation coefficient (MCC); Average F1 score ($\hbox {F1}^a$); F1 score of regression class ($\hbox {F1}^-$); F1 score of the progression class ($\hbox {F1}^+$); Average F1 score ($\hbox {F1}^a$); Mean absolute error (MAE); Mean square error (MSE); Pearson’s correlation coefficient (*r*).

**Table 8:** For all geometric features defined in the original IBIS-4^2^ paper and all clinical features defined at the patient level, the selection count and total mean absolute SHAP values times the selection ratio. Results are presented for the ChPAV and PBR ROI definition criteria.

Feature	ChPAV ROI definition critera		PBR ROI definition criteria	
	SR $\times$ $\Sigma$Mean(|SHAP|)	Sel. Count	SR $\times$ $\Sigma$Mean(|SHAP|)	Sel. Count
$\hbox {PAV}$	5.289	27	146.803	99
$\hbox {TAV}$	34.345	76	0.198	4
$\hbox {TLV}$	0.000	0	0.000	0
$\hbox {TVV}$	0.009	1	0.039	2
$\mathscr {A}$	0.000	1	6.439	84
$\ell$	0.602	9	0.000	0
AGE	0.004	8	0.071	9
MSX	0.000	0	8.286	100
BMI	0.007	6	0.138	13
FHC	0.018	12	1.049	37
DIA	0.003	5	0.026	6
CSK	0.003	6	0.027	6
HYT	0.006	7	0.083	11
HYC	0.008	8	0.018	5
PMI	0.002	14	0.036	7
PPI	0.003	7	0.003	2
STA	0.002	3	0.024	6
RFL	0.001	9	0.068	11
ASP	0.001	2	0.069	11
CLO	0.001	2	0.024	6
PRA	0.003	7	0.236	19
ACE	0.006	9	0.000	1
BB	0.000	3	0.060	9
DAPT	0.000	0	5.118	100
HDL	0.000	0	3.075	100
LDL	0.000	3	0.075	16

## Discussion

### Main contribution

Plaque progression/regression in response to treatment, as quantified from IVUS images, has been the focus of research for more than 20 years. Nevertheless, that research centered primarily on describing the association between treatment and plaque evolution in terms of basic statistics^1,3^. More recently, researchers presented predictive models to assess plaque evolution, see the supplementary material for a summary.

In this work, we presented an XGBoost regressor model to estimate the change in the percent of atheroma volume ($\Delta \hbox {PAV}$) as the end-point, which is the standard measurement of plaque evolution^1^. Previous works did not use such a machine learning model, nor used the $\Delta \hbox {PAV}$, because they center on frame-wise prediction instead of region-wise prediction. We have detailed our pre-processing and training methodology, for which we use 5-Fold cross-validation (20-repetitions) at the patient level, which is considerably more robust than using cross-validation at frame-level since consecutive (and therefore very similar) frames are expected to provide highly correlated data into the training and validation phases. The input data for our method are vessel and lumen contours extracted from IVUS pullbacks, which are already the standard measurement in the clinic, in contrast to other methods that need VH-IVUS, OCT, or 3DQCA and segmentation of other structures such as lipids to obtain morphological features and to construct computational domains for complex computer simulations. Finally, the proposed model was developed with a subset of the IBIS-4 data set, spanning 140 arteries from 81 patients, becoming the largest data set ever used for plaque progression/regression prediction models, compared to state-of-the-art publications which used at most 9 pullbacks.

This work is the first of its kind that aims to contribute to the field through: (i) proposing a complete methodology to process contours extracted from IVUS to generate a comprehensive geometric description; (ii) developing a machine-learning-based model to predict plaque progression/regression from such features and patient data. That is, the main contribution consists in the development of a predictive model to estimate plaque evolution over time, based on information collected at baseline from IVUS images and clinical data.

### Impact of ROI definition

When the full pullbacks were used for model training, i.e. when the FP ROI definition criterion was employed, the models trained with feature sets containing geometric-based descriptors performed poorly. Such a result could be attributed to the fact that full pullbacks in the data set cover different lengths and is common for them to contain regions in which the plaque will increase (progression) and others in which the plaque decrease (regression). The compensation of these regions in the total $\Delta \hbox {PAV}$ is hard to predict when frame-wise features are condensed over the complete pullback. In other words, this confirms the fact that the plaque progression/regression problem depends upon focal phenomena, something that is widely accepted in the specialized literature.

In turn, the FP ROI definition criterion was adequate to test the feature subset $\mathscr {S}^a$, i.e. standalone clinical features defined at the patient level defined in Table 1. Under such a scenario, the method resulted in an average of $0.65~\hbox {ACC}$ and $0.21~\hbox {MCC}$. It is worth noting that such predictive capabilities are similar to those obtained using the ROI definition criterion based on baseline plaque burden alone (PBR). Nonetheless, to correctly interpret such results, the following points should be taken into consideration.

- The FP ROI criterion results in only 140 pullbacks from 81 patients, while PBR ROI criterion resulted in 763 ROIs.
- The feature set $\mathscr {S}^a$ was not used directly on the PBR ROI criterion tests because clinical features are defined at the patient level.
- Comparing FP and PBR criteria on all other feature subsets (including the $\mathscr {S}^a$) resulted in a clear superior performance of the PBR scenarios.
- Importantly, some clinical variables (see Table 8) impacted the predictions of the models based on PBR ROI criterion.

Regarding the ChPAV ROI definition criterion, we present it as a reference for comparison. It highlights that the proposed methodology could reach outstanding prediction capabilities (averages of $0.70~\hbox {ACC}$ and $0.41~\hbox {MCC}$) when the ROIs are homogeneous regarding regression xor progression. It is worth noting that in this scenario, the clinical features portrayed a weak influence on the model’s output (see Table 8). In turn, the PBR ROI definition criterion resulted in averages of $0.60~\hbox {ACC}$ and $0.23~\hbox {MCC}$ for the case $\mathscr {S}^d_{k=32}$, which were the best output among all ROI definition criteria that do not used follow up data.

We performed further tests in which models trained with ChPAV ROIs were used to predict PBR, W30O10, W60O20, and W120O20 ROIs, exploiting the fact that RSKFCV generated folds at the patient level, but prediction metrics were similar to the ones when the same ROIs strategies were used to train/validate in each case. For the sake of clarity, we did not include such results in this manuscript.

Our findings indicate that the locality of the plaque progression/regression problem pointed out above can be tackled using strategies to divide each pullback into ROIs, where geometric features are retrieved using statistics-, information theory- and signal processing-based tools. Of course, the criteria to define these ROIs produce an impact on model performance. This is because of the way in which the homogeneity, the sign, and the zeros of function ${\text {PB}}_{\text {FU}} - {\text {PB}}_{\text{BL}}$ are characterized through each criterion. Indeed, more research should be granted to better delineate ROIs and exploit the information contained in an IVUS pullback to its fullest.

### Predictive value of features

It is worth noting that feature values are intrinsically related to the ROI definition criterion. In Sect. "Impact of ROI definition" we discussed the use of standalone clinical features on the full pullback analysis scenario. We found that clinical features had less predictive power when using the referential ChPAV criterion than when using the PBR criterion. This indicates that the predictive capabilities of these clinical features are moderate to poor (see Table 8). It is plausible that the explanation behind such behavior relies on the number of ROIs per pullback: the ChPAV case generates almost three times the number of ROIs than the PBR case. Since clinical variables are defined at the patient level, then it is expected that they lose predictive power when pullbacks contain regions where plaque either increases or diminishes.

Feature condensation was proved to work better in the ChPAV scenario. This indicates some susceptibility of the condensation methods to the ROI definition criterion. It is possible that condensing a region with multiple progression/regression sub-regions undermines the predictive capabilities of the features defined in this work.

In addition, we showed that performing feature selection improves results. We tested several feature selection methods, e.g. sequential (forward/backward), Boruta^15^, and conditional likelihood maximization^16^. Being the one presented in Sect. "Training/test methodology" the one that resulted in the best predictive metrics.

Adding to this discussion on the feature’s impact on model prediction, it is worth noting that dimensionality reduction techniques are commonly used to transform high-dimensional spaces to a (more human-friendly) low-dimensional representation. We tested the use of Principal Component Analysis (PCA^17^) and Uniform Manifold Approximation and Projection (UMAP^18^). Nevertheless, since performance metrics did not improve, for the sake of clarity such tests were not presented in this manuscript.

Finally, it is important to recall that machine learning algorithms, such as the one proposed in this study, may often lack interpretability, making it challenging to understand the underlying features or the rationale behind their predictions. To gain insight into the role of the features, we used SHAP values^14^ to highlight the feature impact on the model’s prediction outcomes. Such a method could provide clinicians with explanations for the algorithm’s decisions, leading to an overall improvement in trust, acceptance, and adoption in clinical practice.

### Clinical impact

The insidious development of atherosclerosis over years to decades and resultant coronary artery disease increase the risk for the more sudden and dreadful symptomatic variety, acute coronary syndrome (ACS)^19^. Globally, ACS due to plaque rupture and erosion remains a major mortality and morbidity burden for patients. Research has revealed that plaque morphology including but not limited to large necrotic core volumes, plaque burden, thin fibrous caps, and inflammatory mediators define a high-risk plaque prone to rupture and lumen obstruction^20^. Coronary plaque progression and inflammation have thus been a major target for pharmacotherapy and evaluation with intravascular imaging. Primary and secondary prevention with intensive statin therapy has proven effective in reducing clinically significant major adverse cardiac events^21^. Studies such as IBIS-4 (Integrated Biomarkers and Imaging Study-4) (NCT00962416) showed an association between plaque burden regression in non-infarct-related coronary arteries and high-intensity rosuvastatin therapy over 13 months in STEMI patients^2^. Nonetheless, appropriately treated patients on statin therapy are still at risk. Prediction of plaque burden progression among patients and whether a patient would respond favorably to treatment represent elusive major milestones in cardiology. Further, knowing the local regions that would experience progression in advance, one would like to investigate whether local therapies such as drug-eluting balloons can modify the course of the disease.

### Next steps

In this manuscript, we have performed a thorough in-depth analysis of the proposed methodology. We have tested variations on the feature selection strategy and the use of dimensionality reduction techniques (latter not reported). Moreover, we have explored the use of other classification/regression methods, specifically: Nearest Neighbors, Support Vector Machines, Gaussian Process, Fully Connected Neural Networks, Gaussian Naive Bayes, Quadratic Discriminant Analysis, Gaussian Mixture Models, and Linear Regression, not reported in this work. Overall, the XGBoost regressor delivered the best results regarding the metrics presented here. For brevity and clarity, we opted not to present the results of the other models.

The next steps of this research should be oriented towards improving prediction performance by (i) collecting more data and (ii) envisioning a different analysis of ROIs. Regarding the first point, there are two possible courses of action, (i.a) try to access retrospective data of studies similar to IBIS4; or (i.b) design and execute new trials. Regarding the second point, we propose three courses of action, (ii.a) to process frame-wise features as spatial signals (in contrast to ROI-wise features strategies) using models for signal forecasting or natural language processing, such as Recurrent Neural Networks; (ii.b) to explore alternative image-related features from the IVUS pullbacks that could be markers for plaque evolution, such as plaque composition (e.g. image texture, among others), and localization (e.g. bifurcation); and finally, ii.c) to estimate hemodynamic and mechanical environments (through blood flow and structural computer simulations) to gather information on wall shear stresses or plaque inner stresses, and used them as complementary features in the methodology proposed here.

### Limitations

While the machine learning algorithm presented in this study showed promising results in detecting coronary plaque regression or progression from IVUS pullback and standard patient clinical data, it is essential to acknowledge the limitations that should be considered in interpreting and applying the findings presented here.

- Regarding the characteristics of the data, it is known that the performance of any machine learning algorithm heavily relies on the quality and representativeness of the dataset used for training and evaluation. In this context, we highlight the following:
  - All patients were treated with high-intensity statin therapy during the 13-month time window between $\hbox {BL}$ and $\hbox {FU}$ acquisitions. Therefore, the data set may not fully represent the diversity and heterogeneity of lumen, vessel, plaque geometry, and hemodynamic/environmental scenarios that affect atherosclerotic plaque regression and progression mechanisms in different patient populations.
  - The size of the dataset, was deemed adequate for the present pilot study. Nevertheless, the present study must be replicated using a larger, more diverse patient cohort.
- Since lumen and vessel contours used in this work were manually defined by specialized cardiologists, the method’s sensitivity to inter- and intra-observer variability remains to be investigated. Moreover, variability and subjectivity in the annotation process may introduce inherent biases. Furthermore, other intrinsic IVUS image characteristics, e.g. image noise, artifacts, image resolution, gating, and so on, may affect the accuracy of contour delineation, and consequently impact the prediction model. Automatic segmentation of lumen and vessel contours by machine learning algorithms can help to circumvent this issue and increase the number of patients involved in the analysis.
- Although we tackle the interpretability of the model predictions through the incorporation of SHAP values, there is still a considerable number of features, relevant to the proposed methodology, which is not as intuitive as $\{\hbox {PAV}, \hbox {TAV}, \hbox {TLV}, \hbox {TVV}\}$ (those commonly used by cardiologists). This could delay adoption in clinical practice.

In summary, further research and validation efforts are necessary to overcome these limitations and enhance the algorithm’s robustness, generalizability, interpretability, and ethical and regulatory compliance.

### Conclusions

In this work, we presented promising results toward predicting atherosclerotic plaque regression/progression over time from patient data at baseline. Specifically, clinical data was integrated with IVUS-derived data (lumen and vessel contours) at two time points, baseline, and 13-month follow-up, to train an XGBoost regressor/classifier. When such a model is trained/validated on regions defined by the very progression/regression of the plaque burden, the accuracy, and the Mathews Correlation Coefficient were, on average, 0.70 and 0.41 respectively, for stratified k-fold cross-validation ($k=5$, $r=20$, 100 models in total). Using an ROI partition criterion based only on the plaque burden at baseline yielded, on average 0.60 and 0.23 for the accuracy and the Mathews Correlation Coefficient, respectively. The use of fix lengths along the pullback to define ROIs did not improve these metrics.

The proposed framework enables the prediction of plaque changes (positive: progression, and negative: regression) in patients treated with rosuvastatin therapy. Moreover, the method may help to stratify patients at risk of coronary plaque progression, using IVUS images and standard patient clinical data.

### Supplementary Information

Supplementary Information.

## Data Availability

The data-set used during the current study is not publicly available and is part of the IBIS4 Study, https://clinicaltrials.gov/ct2/show/NCT00962416.
