# Supplementary material for: Data-driven models for the prediction of coronary atherosclerotic plaque progression/regression
Source: Sci Rep. 2024 Jan 17;14:1493. doi: 10.1038/s41598-024-51508-7 (PMC10794448; doi:10.1038/s41598-024-51508-7)
Supplement: Supplementary file 1 — Supplementary Information. [file 41598_2024_51508_MOESM1_ESM.pdf]

Supplementary information
Data-driven models for the prediction of coronary atherosclerotic plaque progression/regression

Carlos A.Bulant[1,2], Gustavo A. Boroni[1,2], Ronald Bass[3], Lorenz Räber[4], Pedro A. Lemos[5,6], Héctor M. García-García[3,7,*], and Pablo J. Blanco[8,9,+]

[*]Corresponding author: hector.m.garciagarcia@medstar.net; hect2701@gmail.com
[+]Corresponding author: pjblanco@lncc.br
[1]Instituto PLADEMA, Universidad Nacional del Centro de la Provincia de Buenos Aires (UNICEN), Tandil, Bs.As., Argentina
[2]Consejo Nacional de Investigaciones Científicas y Técnicas (CONICET), Tandil, Bs.As., Argentina
[3]Georgetown University School of Medicine, Washington, DC, USA
[4]Department of Cardiology, Inselspital, Bern University Hospital, University of Bern, Bern, Switzerland
[5]Heart Institute, University of São Paulo Medical School, São Paulo, SP, Brazil
[6]Hospital Israelita Albert Einstein, São Paulo, SP, Brazil
[7]Division of Interventional Cardiology of MedStar Cardiovascular Research Network at MedStar Washington Hospital Center, 110 Irving Street, Suite 4B-1, Washington, DC, 20010, USA
[8]National Laboratory for Scientific Computing (LNCC-MCTI), Petrópolis, RJ, Brazil
[9]National Institute of Science and Technology in Medicine Assisted by Scientific Computing (INCT-MACC), Petrópolis, RJ, Brazil

ABSTRACT

This is the supplementary information document of the main manuscript. It contains a bibliographic review Table of *predictive* models for coronary plaque evolution and details on the implementation parameters for the XGBoost and the Feature Selection methods.

1 Bibliographic review on predictive models for coronary plaque evolution

Ref.	Sample	Image type	End-Point	Features	Predictive Model	Predictive Metrics	Cross-Validation
1	1 patient<br>2 arteries<br>105 frames<br>10 mths FU	IVUS<br>OCT	LAi<br>PAi<br>PBi	14 morph.<br>5 biomech.	LSSVM	LAi ACC=0.911<br>PAi ACC=0.881<br>PBi ACC=0.905	5-fold<br>100-Rep.
2	9 patients<br>9 arteries<br>374 frames<br>8.8±2.6 mths FU	VH-IVUS	MPVIi	7 morph.<br>6 biomech.	RF<br>SVM<br>GLLRM	RF ACC=0.9147<br>SVM ACC=0.9078<br>GLLRM ACC=0.8556	5-fold
3	1 patient<br>2 arteries<br>45 frames<br>10 mths FU	3DQCA<br>IVUS<br>OCT	LPIi<br>CTIi<br>MPVIi	7 morph.<br>6 biomech.	SVM<br>DA<br>RF	SVM LPIi AUC=0.963<br>DA CTIi AUC=0.836<br>RF MPVIi AUC=0.847	5-fold
4	9 patients<br>9 arteries<br>114 frames<br>8.5±1.1 mths FU	IVUS<br>OCT	CTIi	5 morph.<br>4 biomech.	GLMM<br>LSSVM	IVUS+OCT<br>GLMM AUC=0.926, ACC=0.908<br>LSSVM AUC=0.838, ACC=0.757<br>IVUS<br>GLMM AUC = 0.783, ACC=0.746<br>LSSVM AUC=0.780, ACC=0.696	5-fold
5	7 patients<br>7 arteries<br>305 frames<br>9 [6-12] mths FU	VH-IVUS	LAi	7 morph.<br>12 biomech.	RF	ACC=0.8361<br>SEN=0.8625<br>SPE=0.8069	OOB

Table 1. Bibliographic survey of predictive models for coronary plaque evolution. Studies not reporting classification metrics were not included. Abbreviations are as follows: morph.: morphological; biomech.: biomechanical; mths.: months; FU: Follow-up; LAi: Lumen area increase; PAi: Plaque area increase; PBi: Plaque burden increase; MPVIi Plaque Vulnerability Index increment (index proposed by the authors in other publication); LPIi: Lipid percentage index increase; CTIi: Cap thickness index increase.
LSSVM: Least Squares Support Vector Machine; SVM: Support Vector Machines; DA: Discriminant Analysis; RF: Random Forest; GLLRM: Generalized Linear Logistic Regression Model; GLMM: Generalized Linear Mixed Model.
ACC: Accuracy; AUC: Area under the curve; SEN: Sensitivity; SPE: Specificity.
CV: Cross-Validation; OOB: out-of-bag.

2 Computational configuration

The computational implementation was done in `Python v3.12`, with the use of the following packages:

- `numpy`[1] and `pandas`[2] Management of tabular data.
- `scipy`[3] Implementation of statistics and information-based methods, as well as integration and interpolations.
- `scikit-learn`[4] Implementation of repeated stratified k-fold cross-validation, feature selection, and model performance metrics.
- `xgboost`[5] For the implementation of the XGBoost method.
- `shap`[6] For computing SHAP values.

The XGBoost regression was configured as follows:

```
xgboostreg = xgboost.XGBRegressor(
    tree_method: 'auto', scale_pos_weight: 2.0, n_estimators: 256,
```

[1] https://github.com/numpy/numpy
[2] https://github.com/pandas-dev/pandas
[3] https://github.com/scipy/scipy
[4] https://github.com/scikit-learn/scikit-learn
[5] https://github.com/dmlc/xgboost
[6] https://github.com/slundberg/shap

```
    max_depth: 12, learning_rate: 0.125, subsample: 0.95,
    colsample_bytree: 0.9, colsample_bylevel: 0.9, colsample_bynode: 1.0,
    objective: 'reg:squarederror', booster: 'gbtree', use_label_encoder: False,
    eval_metric: 'mae', random_state: 1, verbosity: 1, reg_lambda: 1, reg_alpha: 0)
```

Regarding the feature selection method, a mutual information classification criterion was used, configured as follows:

```
def mutual_information_proxy(X,y):
    return sklearn.feature_selection.mutual_info_classif(
        X,y, discrete_features='auto', n_neighbors=9, proxy = mutual_information_proxy
nfeatures = 32 # Or 8, depending the test case.
proxy = mutual_information_proxy
featsel = sklearn.feature_selection.SelectKBest(proxy, k=nfeatures)
```

References

1. Guo, X. *et al.* A multimodality image-based fluid–structure interaction modeling approach for prediction of coronary plaque progression using IVUS and optical coherence tomography data with follow-up. *J. Biomech. Eng.* **141**, DOI: 10.1115/1.4043866 (2019).
2. Wang, L. *et al.* Using intravascular ultrasound image-based fluid-structure interaction models and machine learning methods to predict human coronary plaque vulnerability change. *Comput. Methods Biomech. Biomed. Eng.* **23**, 1267–1276, DOI: 10.1080/10255842.2020.1795838 (2020).
3. Guo, X. *et al.* Predicting plaque vulnerability change using intravascular ultrasound + optical coherence tomography image-based fluid–structure interaction models and machine learning methods with patient follow-up data: a feasibility study. *BioMedical Eng. OnLine* **20**, DOI: 10.1186/s12938-021-00868-6 (2021).
4. Lv, R. *et al.* Using optical coherence tomography and intravascular ultrasound imaging to quantify coronary plaque cap stress/strain and progression: A follow-up study using 3d thin-layer models. *Front. Bioeng. Biotechnol.* **9**, DOI: 10.3389/fbioe.2021.713525 (2021).
5. Guo, X. *et al.* Predicting coronary stenosis progression using plaque fatigue from IVUS-based thin-slice models: A machine learning random forest approach. *Front. Physiol.* **13**, DOI: 10.3389/fphys.2022.912447 (2022).
